# Discovery of a Novel Cellobiose Dehydrogenase from *Cellulomonas palmilytica* EW123 and Its Sugar Acids Production

**DOI:** 10.4014/jmb.2307.07004

**Published:** 2023-10-05

**Authors:** Ake-kavitch Siriatcharanon, Sawannee Sutheeworapong, Sirilak Baramee, Rattiya Waeonukul, Patthra Pason, Akihiko Kosugi, Ayaka Uke, Khanok Ratanakhanokchai, Chakrit Tachaapaikoon

**Affiliations:** 1 Division of Biochemical Technology, School of Bioresources and Technology, King Mongkut's University of Technology Thonburi (KMUTT), Bangkok 10150, Thailand; 2 Division of Bioinformatics and Systems Biology, School of Bioresources and Technology, King Mongkut's University of Technology Thonburi (KMUTT), Bangkok 10150, Thailand; 3 Excellent Center of Enzyme Technology and Microbial Utilization, Pilot Plant Development and Training Institute, King Mongkut's University of Technology Thonburi (KMUTT), Bangkok 10150, Thailand; 4 Biological Resources and Post-harvest Division, Japan International Research Center for Agricultural Sciences (JIRCAS), 1-1 Ohwashi, Tsukuba, Ibaraki 305-8686, Japan

**Keywords:** Actinobacterium, cellobionic acid, cellobiose dehydrogenase, *Cellulomonas palmilytica*, lactobionic acid

## Abstract

Cellobiose dehydrogenases (CDHs) are a group of enzymes belonging to the hemoflavoenzyme group, which are mostly found in fungi. They play an important role in the production of acid sugar. In this research, CDH annotated from the actinobacterium *Cellulomonas palmilytica* EW123 (*Cp*CDH) was cloned and characterized. The *Cp*CDH exhibited a domain architecture resembling class-I CDH found in Basidiomycota. The cytochrome c and flavin-containing dehydrogenase domains in *Cp*CDH showed an extra-long evolutionary distance compared to fungal CDH. The amino acid sequence of *Cp*CDH revealed conservative catalytic amino acids and a distinct flavin adenine dinucleotide region specific to CDH, setting it apart from closely related sequences. The physicochemical properties of *Cp*CDH displayed optimal pH conditions similar to those of CDHs but differed in terms of optimal temperature. The *Cp*CDH displayed excellent enzymatic activity at low temperatures (below 30°C), unlike other CDHs. Moreover, *Cp*CDH showed the highest substrate specificity for disaccharides such as cellobiose and lactose, which contain a glucose molecule at the non-reducing end. The catalytic efficiency of *Cp*CDH for cellobiose and lactose were 2.05 x 10^5^ and 9.06 x 10^4^ (M^–1^ s^–1^), respectively. The result from the Fourier-transform infrared spectroscopy (FT-IR) spectra confirmed the presence of cellobionic and lactobionic acids as the oxidative products of *Cp*CDH. This study establishes *Cp*CDH as a novel and attractive bacterial CDH, representing the first report of its kind in the *Cellulomonas* genus.

## Introduction

Cellobiose dehydrogenases (CHDs) (EC 1.1.99.18) or cellobiose 1-oxidoreductases are hemoflavoenzymes that are mostly found in fungi, specifically in Basidiomycota and Ascomycota. They belong to the glucose-methanol-choline (GMC) superfamily of oxidoreductases, based on sequence similarity [1]. All members of the GMC superfamily share a common protein architecture, with flavin adenine dinucleotide (FAD) as a cofactor. The catalytic domain is highly conserved, with the catalytic His/Asn residues found in the active site [2-4]. In the CDH protein structure, the N-terminal is presented with an electron-transferring cytochrome (CYT) domain. This domain interacts and works with other cellulolytic and hemicellulolytic enzymes to enhance cellulose or hemicellulose depolymerization [5-7]. Based on previous findings, CDH acts as an electron donor for lytic polysaccharide monooxygenase (LPMO) in a synergistic manner [8], in which LPMO plays an important role as crystalline-cellulose breaker. The catalytic or dehydrogenase domain (DH) is located at the C-terminus, and certain CDHs also possess carbohydrate-binding module (CBM) family 1 on the C-terminus of the DH domain [9]. Based on phylogeny analysis, CDH has been classified into four classes: I to IV. Class I is found exclusively in Basidiomycota, whereas Classes II, III, and IV are found in Ascomycota [9, 10]. The CDHs were classified based on their amino acid sequences and protein architectures. A comprehensive sequence-based classification system on carbohydrate-active enzymes database (CAZy) is continuously updated, followed by the growing availability of resource data (*e.g.*, whole genome sequence database) [11]. Within this classification, CDH is placed in the auxiliary activity (AA) category, specifically in family AA3. The AA3 is further divided into four subfamilies based on substrate specificity, which includes AA3_1 (cellobiose dehydrogenase), AA3_2 (aryl-alcohol oxidase, aryl-alcohol dehydrogenase, glucose oxidase, glucose dehydrogenase, and pyranose dehydrogenase), AA3_3 (alcohol oxidase), and AA3_4 (pyranose oxidase). The biological function of CDH is widely described, such as substrate-inhibition removal for cellulase by converting cellobiose to cellobionic acid, as electron donor for lignin-degrading enzymes and other putative activities [12, 13]. In addition, CDH can be applied in sugar acid production *e.g.*, cellobionic and lactobionic acids [14, 15]. Various applications of cellobionic and lactobionic acids have been reported, and they are extensively used in various fields, including as antioxidants, carcinogen reducers, and antimicrobial agents in the food industry, as well as in cosmetics and pharmaceuticals. Furthermore, lactobionic acid has demonstrated potential as an ingredient in organ preservation solutions in medical applications [16, 17].

Recently, we successfully isolated a highly efficient microorganism for oil palm empty fruit bunch (OPEFB) degradation, a novel species termed *Cellulomonas palmilytica* EW123 [18]. During the period of enzymatic activity and cellulose saccharification, a low concentration of cellobiose was present in the *C. palmilytica* EW123 culture broth when growing on OPEFB (unpublished data). It is hypothesized that these compounds are converted into other compounds, such as acid sugars, through the activity of CDH. Furthermore, genome annotation revealed the identification of one CDH annotation (WP_236123495) with a low BLAST score. In this study, the gene encoding the CDH enzyme of *C. palmilytica* EW123 was cloned and expressed in *E. coli* BL21 (DE3). Subsequently, the recombinant CDH was characterized and found to be capable of producing cellobionic and lactobionic acids from cellobiose and lactose, respectively. This represents a novel discovery of CDH from the bacterial genus *Cellulomonas*, which has been characterized and reported.

## Materials and Methods

### Bacterial Culture, Gene Cloning, and Protein Purification

Strain *C. palmilytica* EW123 was cultured in modified B medium with the following composition (g/l):(NH_4_)_2_SO_4_ 0.5, K_2_HPO_4_ 6.5, KH_2_PO_4_ 3.5, CaCl_2_·2 H_2_O 0.05, CuSO_4_·5 H_2_O 0.05, MgSO_4_·7 H_2_O 0.05, MnSO_4_·H_2_O 0.05, NaCl 1.0, yeast extract 0.1, and D-cellobiose 5.0 (Sigma-Aldrich, USA) as sole carbon source [19]. For *E. coli*, DH5a and BL21 (DE3) were cultured in LB medium. The chromosomal DNA was extracted using the DNeasy blood & tissue kit (Qiagen, USA), in which plasmid and DNA fragments were extracted and purified using the QIAprep spin miniprep, QIAquick PCR purification kit, and QIAquick gel extraction kit (Qiagen). The CDH gene was amplified via a nested PCR using two couple primers: N-PCR_F 3’-CGCTTCTCCATGACTCGGTA-5’, N-PCR_R 3’-CCTCCACGCTAAGAACCGTC-5’, *Cp*CDH_F 3’-CATATGGTCGAGCCGCGCCAC-5’, and *Cp*CDH_R 3’-GCTGAGCTCACATCTCCCCCACGT-5’. The nested-PCR primers were designed by Primer-BLAST on the NCBI web service [20]. The PCR fragment was amplified by touchdown PCR (TD-PCR) to increase specificity with an initial higher temperature. The amplification condition was 10 TD-PCR cycles of 30 s at 95°C, 30 s at Ta (-1.2°C/cycle), and 20 standard cycles (Table S1). The Q5 high-fidelity DNA polymerase (NEB, UK) with deoxynucleotide mix (G+C: A+T is 72:25 molar ratio) was used along the amplification procedure. Commercialized plasmid pET-28a was applied to carry out CDH gene expression. The recombinant CDH was purified via the HisTrap HP column with ÄKTA pure (GE Healthcare, USA), whose protein content was demonstrated by OD_280_ and the Bradford protein assay (Bio-Rad, USA). The purified protein was analyzed via the blue native polyacrylamide gel electrophoresis (PAGE) principle [21], and the protein was stained employing the InstantBlue coomassie protein stain (Abcam, UK). The in-gel enzymatic activity was modified as follows: zymography was performed in native PAGE that stained with 25 mM 2,6-dichlorophenol indophenol (DCPIP) in 100 mM sodium acetate buffer pH 5.0, and enzymatic activity was initiated by soaking the gel in 200 mM lactose [22]. The water for the enzymatic reaction was saturated with oxygen gas (purity at 99.8%) by sparging.

### The *cdh* Gene and Phylogeny Analysis

The *cdh* gene sequence (WP_236123495 in NCBI database) was retrieved from *C. palmilytica* EW123 genome GenBank, Accession CP06222, on the NCBI database under BioProject number PRJNA564365. Both nucleotide and amino acid sequences were determined using BioEdit 7.2 [23] and MEGA 11.0 [24] programs. The protein molecular mass (Mw) and the isoelectric point (pI) were calculated with the Expasy web interface services [25, 26]. The phylogeny was analyzed and done via maximum likelihood (ML) algorithms with 1,000 bootstrap replications. The BlastP suit service on NCBI was employed for sequence BLAST, and the obtained sequences were aligned by Clustal Omega on the web service interface [27]. The protein secondary structure was predicted using the deep learning extension of the convolutional neural field or DeepCNF on the RaptorX-Property web server (http://raptorx2.uchicago.edu/StructurePropertyPred/predict/) [28].

### CDH Activity Assay and Electron Acceptor Study

The activity of CDH was measured using a spectrophotometric assay that detected the reduction of DCPIP at 520 nm. The assay was carried out with 15 μM CDH, 300 μM DCPIP, 30 mM cellobiose, and 50 mM sodium acetate buffer pH 5.0 for 5 min at 25°C. Enzymatic activity was defined as the amount of enzyme that could oxidize 1 μM of the electron acceptor (DCPIP) per minute under the assay conditions. The extinction coefficient (ε) of DCPIP was 6.8 mM^–1^ cm^–1^, and the absorption coefficient values differed by less than 3% across the entire pH range [29]. The activity of CDH was studied under different conditions, including pH, temperature, and substrate specificity, using the assay described above. To examine the electron acceptors, both ABTS (ε = 36 mM^–1^ cm^–1^ at 420 nm) and DCPIP were employed.

### CDH Kinetic Measurement

The enzyme kinetics were investigated under assay conditions with varying substrate concentrations ranging from 0 to 300 mM at the optimal pH and temperature conditions. The electron donors, including cellobiose, lactose, and glucose, were examined [30].

### Oxidative Product of CDH Determination

Lactobionic acid from enzymatic reactions was analyzed using HPLC (LC-2014; Shimadzu, Japan) compared with standard lactobionic acid (Sigma-Aldrich, USA). The HPLC conditions were as follows: 30% acetonitrile and 5 mM sulfuric acid as mobile phase with a flow rate at 0.4 ml/min under 40°C oven temperature, with Aminex HPX-87H (Bio-Rad) as the performing column. Cellobionic acid was analyzed with FT-IR (Perkin-Elmer Spectrum Two, UK) to determine the different functional group on the sugar acid structure. The spectral range was 4,000–550 cm^–1^, which was analyzed with the Spectrum 10 software. To purify cellobionic and lactobionic acid, Type I strong anion exchange resin (DIAION SA10 Series, Mitsubishi, Japan) was used and eluted with a series of HCl concentrations (0.1 to 1.0 M). Prior to analysis, the eluent fractions were collected and dried using a freeze dryer (Gold-Sim, FD5-2.5E).

## Results

### The *cdh* Gene Analysis and Phylogenetic Tree

The *cdh* gene from *C. palmilytica* EW123 was identified, analyzed and termed *Cp*CDH. It consisted of 2,769 bp and was translated into a sequence of 923 amino acid residues, starting with alanine followed by 30 residues that served as a signal peptide of the Sec system to translocate unfolded proteins across the cytoplasmic membrane. The signal peptide segment of *Cp*CDH was excluded from cloning and expression under the gene-specific amplification step. The theoretical pI and Mw values were 10.89 and 94,956.54 Da, respectively. The protein homology search using HMMER indicated that *Cp*CDH contained two domains: the CYT domain on the N-terminus and DH domains on the C-terminal sequence. The BLAST analysis conducted on the NCBI database identified *Cp*CDH as a member of the GMC oxidoreductase superfamily. However, the query cover only reached 85%, and the identity matrix did not exceed 88% when compared to that of a non-characterized database. The query cover value suggested the presence of regions in *Cp*CDH that differ significantly from those in the database, whereas the identity matrix indicated a similarity in the compared regions. These parameters were assessed using a non-redundant database. For further analysis, a characterized database in the AA3 family was used, and the result showed that the amino acid sequence of *Cp*CDH was only 21.96% identical to that of the characterized CDH enzymes in the GMC superfamily. Subsequently, the ML phylogenetic tree of the *Cp*CDH amino acid sequence was generated with 1,000 replications. The analysis indicated that *Cp*CDH belonged to the AA3 family but differed significantly from the other enzymes. In fact, the location of *Cp*CDH in this phylogeny was distinct from that of other clads, and the number of substitutions between *Cp*CDH and other CDH enzymes ranged from 12.4 to 13.58.

**Fig. 1:**
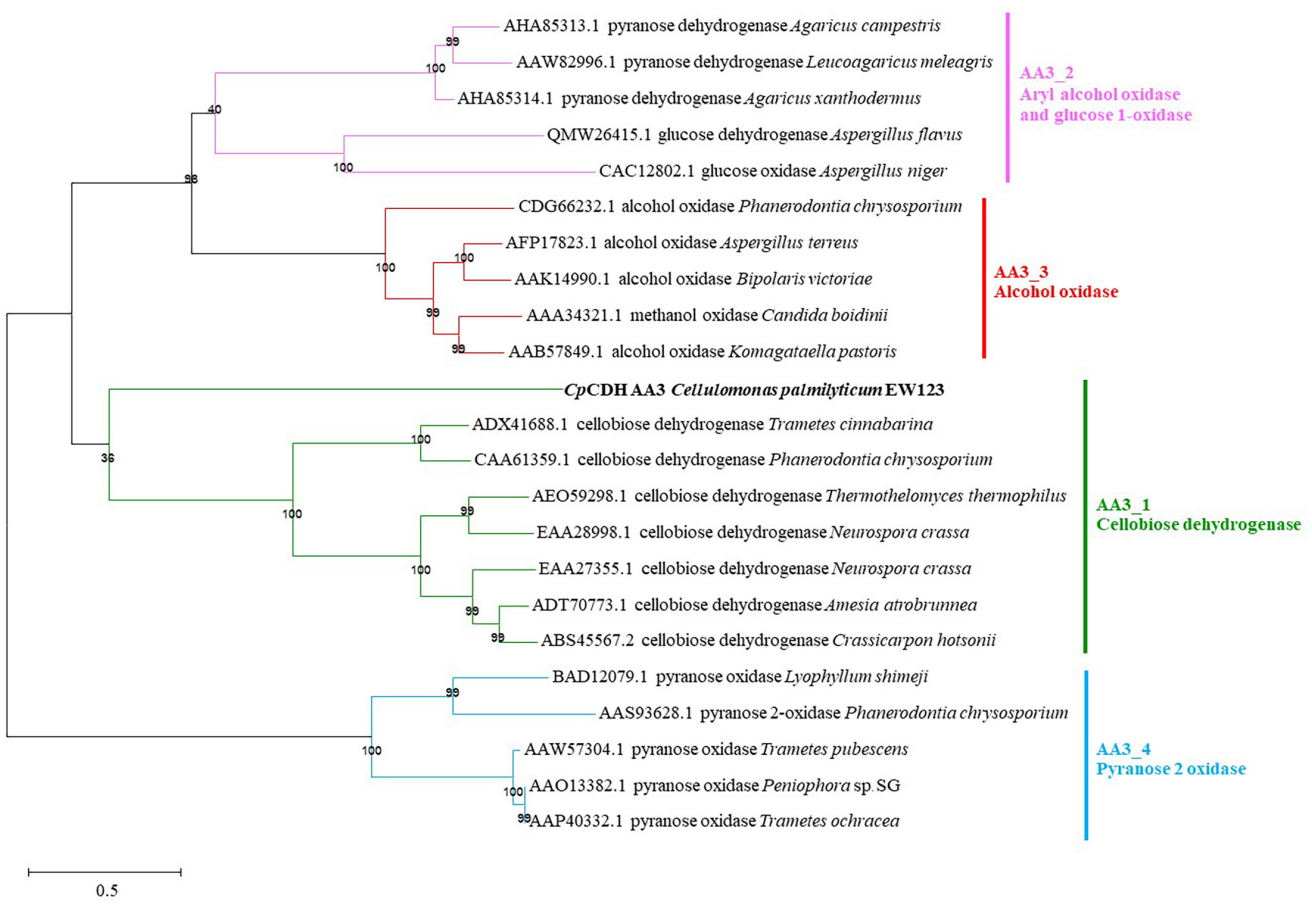
The phylogeny analysis of *Cp*CDH and other characterized GMC oxidoreductases from AA3 family includes 25 sequence subjects from four subfamilies that present in different colors.

**Fig. 2:**
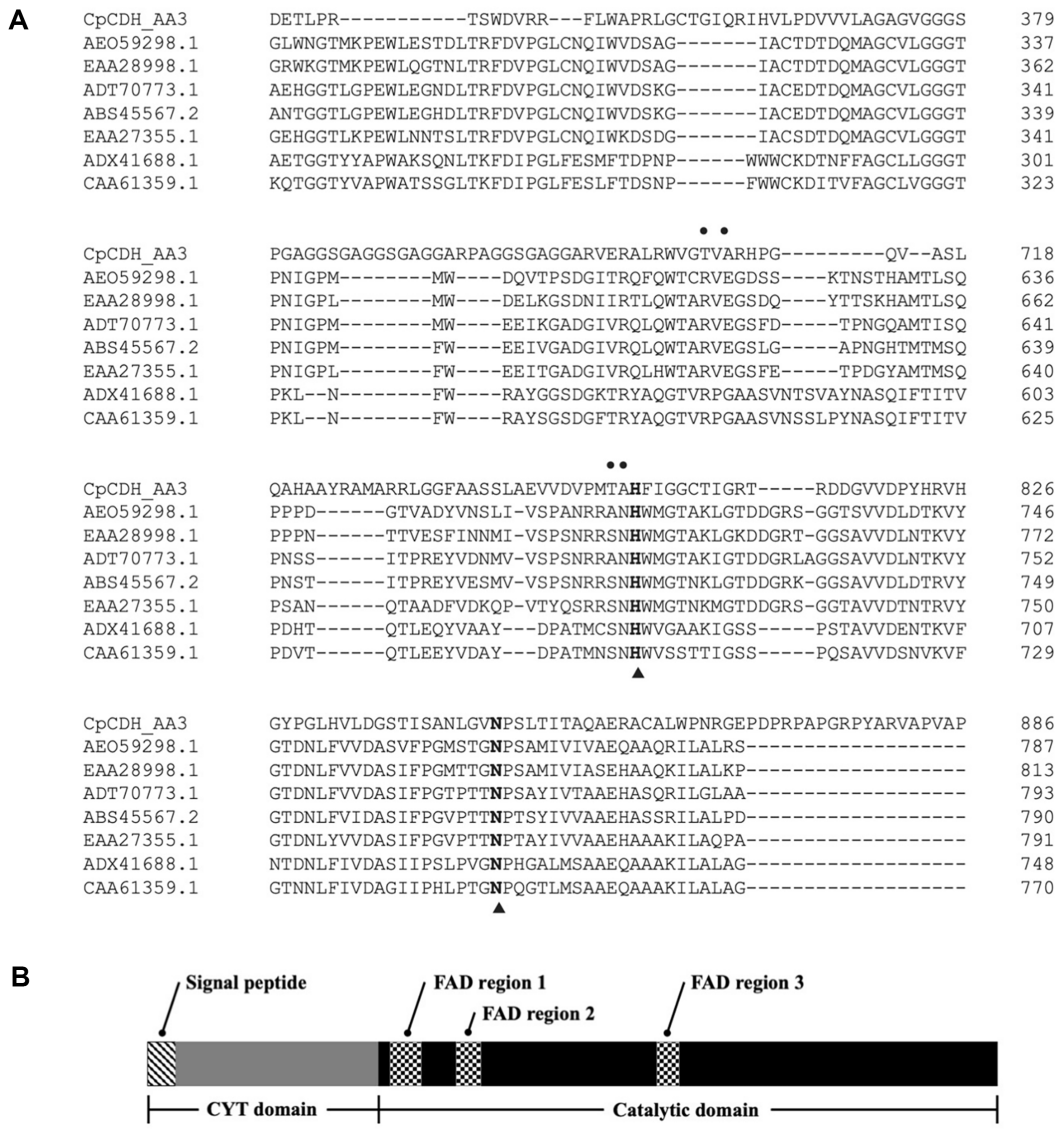
The informative illustration of *Cp*CDH amino acid sequence. (**A**) the schematic illustration of *Cp*CDH. (**B**) a part of multiple sequence alignment of *Cp*CDH with other CDHs. The catalytic residues were presented with triangle under amino acid sequence and binding amino acid residues were indicated with bold circle above the amino acid sequence.

Fig. 1 shows the phylogenetic tree of *Cp*CDH. Multiple sequence alignment of *Cp*CDH with GMC oxidoreductase from the AA3 family was performed (Fig. S1), and a part of the sequence alignment with CDH is shown in Fig. 2A. Fig. 2B shows the schematic illustration of *Cp*CDH. The *Cp*CDH shared an amino acid sequence that was closely related to that of the CDHs enzymes, with focus on the DH domain. The conserved FAD binding regions are indicated in boxes (Fig. S1), and catalytic residues are indicated with solid triangle symbols in both Figs. 2A and S1; the predictive catalytic amino acids were His^803^ and Asn^846^. The full sequence analysis of the multiple sequence alignment of the AA3 family is shown in Fig. S1.

### The *cdh* Gene Cloning and Expression

Both nested and *cdh* PCR fragments were successfully amplified, and the PCR products are presented with 3,823 and 2,772 bp, respectively (Fig. S2). The *cdh* gene was cloned into vector plasmid pET-28a between *NdeI* and *NotI* sites. To discard a C-terminal histidine tag, the termination codon sequence was directly added into the reverse primer sequence. The purified recombinant *Cp*CDH is illustrated in Fig. S3. Fig. S3 shows a single band (lane 1), which has a molecular mass of 97.89 kDa, as observed in the gel filtration chromatography (Fig. S4). The in-gel enzymatic activity was investigated in native-PAGE, as indicated in Fig. S3 (lane 2).

### Biochemical Properties and Enzyme Kinetics of the Recombinant *Cp*CDH

**Fig. 3:**
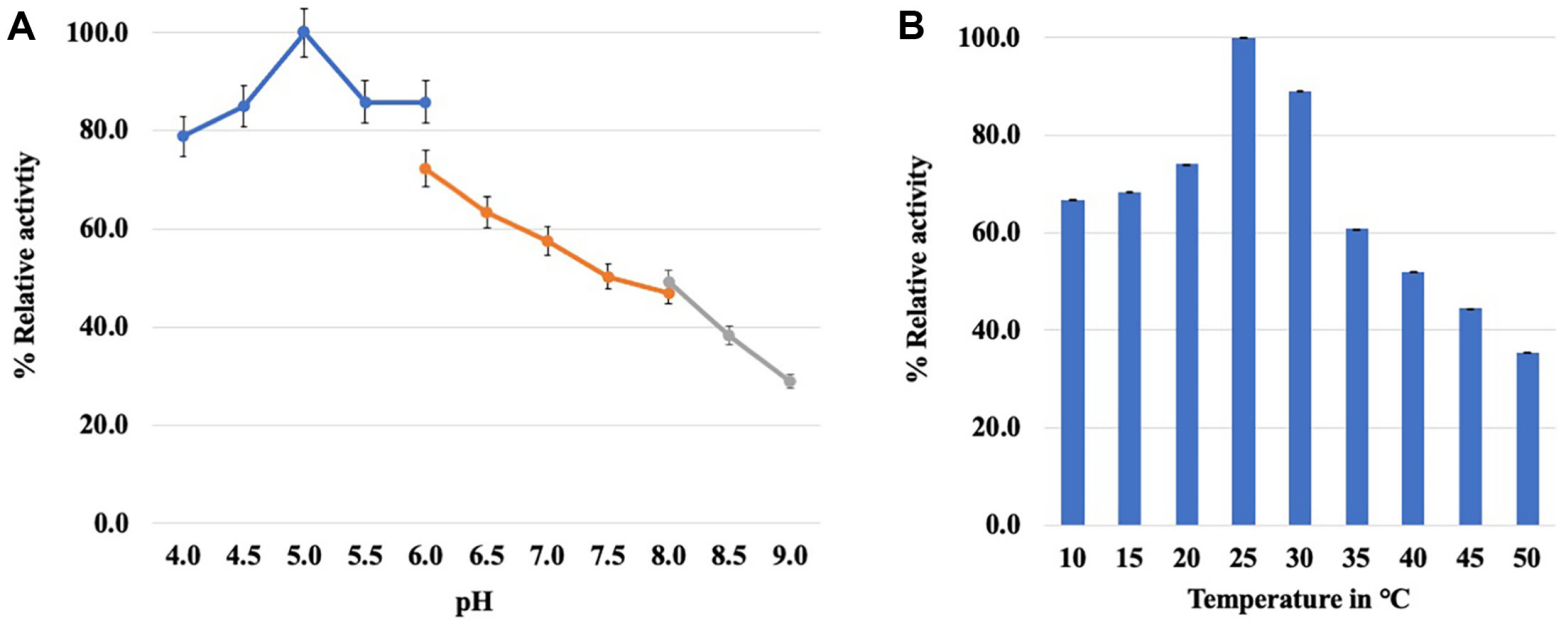
The percentage relative activity of *Cp*CDH in mocking condition assay. (**A**) optimal pH within three buffer systems (acetate buffer for 4.0-6.0, phosphate buffer for 6.0-8.0 and Tris-HCl for 8.0-9.0) and (**B**) an optimal temperature in degree Celsius unit.

Fig. 3 shows the relative values of the optimal pH and temperature of recombinant *Cp*CDH. The pH optimum was 5.0 (Fig. 3A) with sodium acetate buffer, and the optimal temperature 25°C (Fig. 3B) under the assay conditions. The substrate specificity of recombinant *Cp*CDH was investigated with mono-, disaccharides with various types of sugars, and results are shown in Table 1. In addition, the glucose containing substrates or related isoform substrates were tested, and the result showed that the *Cp*CDH had slight activity with maltose (< 0.005 unit/ml) and devoid activity toward sucrose, mannobiose, and xylobiose. Cellulose polymers were also examined, and no enzymatic activity was observed toward both cellulose powder and phosphoric acid swollen cellulose. The oxidizing agents for electron transfer were investigated, and ABTS and DCPIP exclusively acted as mediators for the recombinant *Cp*CDH. The DCPIP showed a higher performance than ABTS, around 10.7-fold with cellobiose and 9.9-fold with lactose. The steady-state kinetics constants for cellobiose, lactose, and glucose were elucidated using artificial electron acceptors such as DCPIP, as shown in Table 2. The Michaelis-Menten plots were generated for each substrate at the micromolar level, demonstrating that the catalytic efficiency of recombinant *Cp*CDH toward cellobiose was higher than that towards lactose and glucose. The catalytic efficiency (*k*_cat_/*K*_m_) for each substrate relative to cellobiose was 2.26- and over 1,400-fold for lactose and glucose, respectively. A low catalytic efficiency was observed for glucose, with a high *K*_m_ value of more than 2,500-fold that of cellobiose, indicating that glucose is a poor substrate for recombinant *Cp*CDH. The catalytic efficiency of *Cp*CDH was closely related to that of other CDHs, such as *Pc*CDH, *Cs*CDH, and *Sr*CDH. These CDHs revealed a higher *k*_cat_/*K*_m_ value toward cellobiose than lactose. However, *Nc*CDH showed a slightly different catalytic efficiency value for both substrates (Table 2).

### The FT-IR Spectra of Purified Sugar Acids

**Fig. 4:**
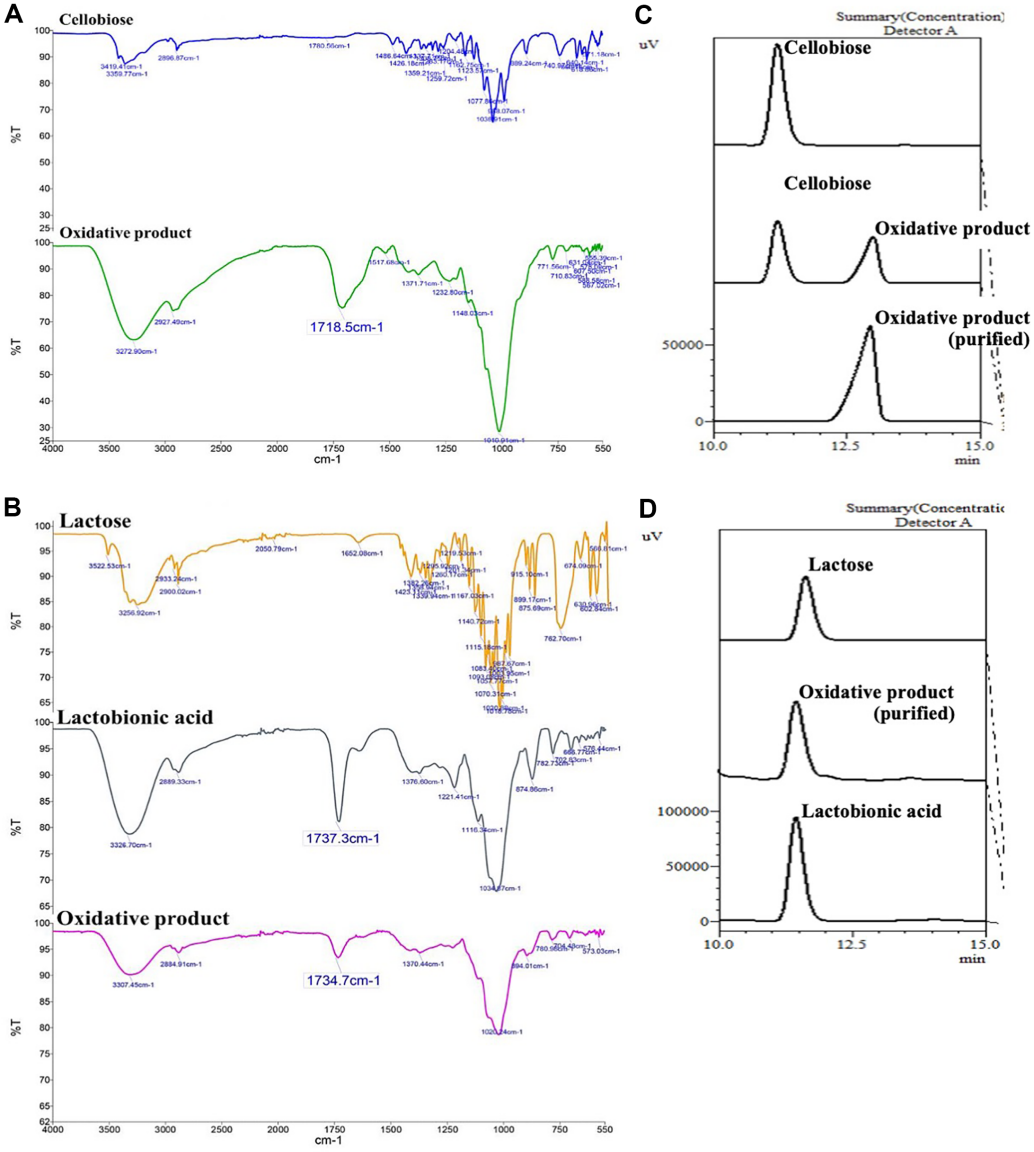
The FT-IR and HPLC chromatogram of substrate and oxidation products are presented. Purified oxidative product of cellobiose (**A**) and lactose (**B**) that were used in FT-IR analysis on the left, while HPLC analysis was presented on the right; purified oxidative product from cellobiose (**C**) and lactose (**D**), respectively.

After the reaction between the recombinant *Cp*CDH with cellobiose and lactose, the resulting oxidative products were purified, and the functional groups were analyzed. The purification of cellobionic and lactobionic acid was successfully achieved using 60- and 40- mM hydrochloric acid as eluent for cellobionic and lactobionic acid, respectively. The purified cellobionic and lactobionic acids were then analyzed to determine the presence of crucial functional groups by FT-IR. The FT-IR chromatogram is presented in Fig. 4. This result clearly indicates that cellobionic and lactobionic acids are the oxidative products of cellobiose and lactose, respectively, as they contain a dominative carboxylic acid group in their structures. The chromatograms showed a narrow absorption peak corresponding to the C=O stretching of unconjugated carboxylic acid groups in the double bond region (1,500–2,000 cm^–1^), specifically around 1,700–1,725 cm^–1^. However, it is worth noting that the chromatogram for carboxylic acids was not included for both the cellobiose and lactose substrates. Additionally, the key absorption in the basic structure of both sugars and the oxidative products are presented. The chromatograms show the O-H stretching of the hydroxyl group in the single bond region (2,500–4,000 cm^–1^), characterized by a broad absorption band between 3,570 and 3,200 cm^–1^. Furthermore, the chromatograms revealed signals corresponding to the C-O stretching of primary alcohols at approximately 1,050 cm^–1^ as well as hydroxyl bending (C-O-H) in the range of 1,440–1,395 cm^–1^.

## Discussion

Generally, *cdh* genes are widely distributed in wood-degrading fungi, both in Ascomycota and Basidiomycota, but have not been reported for bacterial CDH [9, 31]. The *Cp*CDH is the first demonstrated CDH enzyme in the genus *Cellulomonas*, derived from an effective oil palm residue degrader, *C. palmilytica* EW123. The amino acid sequence of *Cp*CDH is notable for its large size and unique pI value. According to a previous study, the largest CDH chain length is found in CDH from the rice blast fungus *Magnaporthe grisea* (XM_360402), with 840 amino acid residues [10]. In contrast, *Cp*CDH contains 923 amino acid residues, with the CYT domain being the primary domain responsible for the higher amino acid chain length. Specifically, the CYT domain of *Cp*CDH (including the linker region) is approximately 300 amino acids, whereas other CDHs typically contain around 200 amino acids. The average pI values of CDH enzymes range from acidic to neutral, between 4.0 and 7.9 [10]. However, *Cp*CDH is uniquely illustrated as having a pI value of 10.89. Upon deconstructing the sequence for focused analysis, the pI value of the CYT domain was ascertained to be 11.65, characterized by a scarcity of conserved amino acids essential for heme binding. In contrast, the DH domain displayed a pI value of 9.86. The theoretical pI was calculated from the total negatively and positively charged residues the from amino acid sequence, which were calculated by Asp+Glu and Arg+Lys, in the sequent [32, 33]. Additionally, the amino acid composition of *Cp*CDH contained arginine, histidine, and lysine at 11.3%, 2.1%, and 0.3% (Table S2), respectively. It should be noted that the number of negatively charged residues in the *Cp*CDH amino acid sequence is 64 residues lower than that of positively charged residues (107 residues) (Table S2), which led to a calculated theoretical pH for *Cp*CDH that indicates basicity and was measured at 10.89. Furthermore, the domain structure of *Cp*CDH is similar to that of Class-I CDH from Basidiomycota such as *Ceriporiopsis subvermispora* (*Cs*CDH), consisting of a CYT domain followed by a DH domain [34]. The signal peptide motif of Sec was located at the N-terminus, followed by the CYT domain from amino acid 31 to Leu^266^, which was connected to 32 residues long of the linker (Thr^267^ to Ala^298^). The C-terminus consisted of 625 amino acid residues of the DH domain, with no significant presence of the CBM1 domain as indicated by the BLAST result. The domain architecture of *Cp*CDH is identical to that of Class-I CDH by the absence of a CBM1 domain, unlike in Class-II CDH [5, 9].

To gain a better understanding of the evolution of *Cp*CDH from *C. palmilytica* EW123, a phylogenetic tree was used for exploration. Phylogenetic analysis within the AA3 family suggested that *Cp*CDH belongs to the CDH group as it shared the same clad (Fig. 1). However, there was a considerable difference in amino acid substitution between *Cp*CDH and other characterized CDHs, ranging from 12.40 to 13.58. Evolutionary analysis suggested that the bacterial *Cp*CDH might have diverged from an ancestor in the evolution line a long time ago, as indicated by the branch length between *Cp*CDH and other fungal CDHs. Nevertheless, the evolutionary route of fungal CDHs is still under investigation, and it is suggested that CDH sequences from the most primitive phyla Chytridiomycota and Zygomycota might fulfill the evolutionary line [31]. To consider the evolutionary line of CDHs, the primary CDHs from the bacteria might also be part of the evolutionary database. Thus, these results suggest that *Cp*CDH might be one of the pioneering CDH enzymes in the bacterial phyla.

To expand our insight into the differences between *Cp*CDH and other CDHs, a comparative analysis was performed. The multiple sequence alignments of *Cp*CDH with other CDH enzymes showed the conservative nature of the FAD binding region and the key catalytic residues. In particular, the catalytic residue of *Cp*CDH was identified as His^803^ and Asn^846^, which are essential for CDH activity [35, 36]. Previous studies have suggested that His^803^ in *Cp*CDH plays a role in base-assisted deprotonation of the C1 hydroxyl group, leading to the formation of a substrate carbanion [36]. Additionally, Asn^846^ has a dual function in CDH enzymes, including substrate positioning and promoting flavin structure, as well as providing hydrogen bonding to O1 and facilitating proton abstraction by His^803^. When comparing the homology model, it was found that the Asn^846^ on *Cp*CDH corresponds to Asn^688^ in *Phanerochaete chrysosporium* (*Pc*CDH) [35] and Asn^700^ in *Myriococcum thermophilum* (*Mt*CDH)[37], indicating that Asn^846^ plays a role in influencing both substrate and oxygen turnover. In addition, Val^527^ in *Cp*CDH, which corresponds to Val^463^ in *Neurospora crassa* (*Nc*CDH), is important for binding to amide nitrogen on nitrogenous base adenine in the FAD structure [38]. Another vital region for the DH domain is the FAD binding site, which is generally found in three regions, as illustrated in Fig. S5. The conserved FAD binding regions were present in boxes. The FAD binding region includes the unique characteristic of Rossmann-fold (βαβ-motif), which is frequently found in both FAD and NAD binding regions [9]. The first FAD binding region was elucidated and found to play a role in ribose sugar binding on the hydroxyl group with glucose residue in CDHs. Multiple sequence alignment showed that Glu^327^ (in position 300-333) of *Cp*CDH collaborated with Glu^282^ in *Nc*CDH (position 255–288), as illustrated in Fig. S1.

Sequence alignment revealed that the FAD region on *Cp*CDH differs significantly from that of other comparators, with identities from only 42.42% to 48.48%. Moving on to the second FAD binding region, this region was found to stabilize the FAD backbone by binding to the amide nitrogen and phosphate group during catalysis. The percentage similarity of this region on *Cp*CDH to that of other comparators was between 30.77 to 34.62 (Fig. S5). Finally, the FAD binding region maintained FAD cavities that bind to FAD [38]. Although this region has the shortest chain length, it still shows an identity matrix with 31.82% to 40.91% (Fig. S5). Despite the low percentage of similarity, the key amino acids in each region were aligned and illustrated identically. In fungal CDH cases, Basidiomycetes and Ascomycetes showed an identity matrix of approximately 65% to 83% within the same phyla and between 30% and 35% when compared across phyla [10, 31]. Thus, the FAD regions of *Cp*CDH clearly demonstrate its novelty when compared to other fungal CDH enzymes.

The CYT domain, situated at the N-terminus of CDH, holds significant importance as it serves as an alternative electron acceptor. This domain facilitates the transfer of electrons to external acceptors, including electrode surfaces or other electrodes [8, 39, 40]. Based on the multiple sequence alignment, the CYT domain from *Cp*CDH has a homology different from that of other subject sequences as it lacks the essential residues Met and His that bind to ferrous ion in heme (Fig. S1). In contrast, fungal CYT domains such as *Nc*CDH [38] and *Pc*CDH [35] exhibit conserved Met and His amino acids that play a role in heme binding [41]. Although the CYT domain can assume a variety of protein structures [31], the structure of the CYT domain in fungal CDHs is conserved and easily observed (Fig. S1). However, the CYT domain of *Cp*CDH was studied as the secondary structure using DeepCNF, and only a small fraction of the CYT domain (17.67%) was identified as having α-helix and β-sheet structural protein, with the majority (82.33%) in the coil and loop form (data not shown). Further examination of the cytochrome domain of basidiomycetes and ascomycetes, using an inferred unroot phylogenetic tree, revealed that the cytochrome node from both phyla is distinctly separated, with an average sequence identity of only 44.5%[31]. Furthermore, the evolutionary route of the fungal and actinobacteria cytochrome c domain diverged significantly, with a considerable evaluation distance [42]. Thus, the extremely low identity matrix of *Cp*CDH, which was first discovered in actinobacteria such as *Cellulomonas*, may be due to the separation of bacterial and fungal ancestors long ago or unexpected incidents such as horizontal gene transfer between kingdoms or phyla. Nonetheless, further investigations of bacterial CDHs are necessary to obtain more information and complete the CDH evolutionary route.

Since basic biochemical properties are essential for potential applications, an investigation into the biochemical properties of the recombinant *Cp*CDH was conducted. The pH optimum of *Cp*CDH was 5.0, which is consistent with other reports of fungal CDHs operating optimally under acidic conditions [10]. However, the cellobiose dehydrogenase from the ascomycete *Humicola insolens* (*Hi*CDH) showed optimal activity under slightly alkaline conditions [43]. The average optimal pH for CDHs under acidic conditions may be due to key amino acid residues in the DH domain. In contrast, the optimal temperature for the recombinant *Cp*CDH was 25°C (with 89.1% at 30°C), which is in contrast to previous findings [10]. The optimal temperature of *Cp*CDH is expected to be similar to its growth-temperature profile, indicating that it can grow within a temperature range of 5 to 37°C within 1 day. However, growth at temperatures lower than 15°C requires 2 days (data not shown). The optimal temperature of *Cp*CDH is opposite to that of fungal CDHs such as *Trametes versicolor* (*Tv*CDH), *Cs*CDH, and *Pc*CDH, which exhibit optimal activity at high temperatures (50–70°C) [22, 34, 44]. The prime operational efficiency of *Cp*CDH is evident within the pH range of 5.0 to 6.0. During catalysis, the amino acids crucial to *Cp*CDH's functionality (H^803^ and Asn^846^) exhibit identical characteristics as those found in other previously reported CDHs. This congruence underscores the necessity for designing optimal enzymatic activity in accordance with this established trend. The variance in pI values between *Cp*CDH and its counterparts may potentially be linked to enzymatic stability or post-translational modifications specific to *C. palmilytica* EW123. Noteworthy is the unpublished insight that the extracellular enzyme originating from *C. palmilytica* EW123, including CDH, exhibits traits of a glycosylation-type protein, as evidenced by positive staining with the periodic acid-Schiff (PAS) method. Based on our results, DCPIP is approximately 8.2-fold more suitable as an electron acceptor compared to ABTS. This observation can be attributed to the difference in redox potentials between the two electron acceptors. The redox potential of DCPIP was approximately +130 mV, whereas those of ABTS were +472 and +885 mV. Therefore, due to its lower redox potential, DCPIP functions more effectively as an electron acceptor than ABTS.

To gain insights into the function of *Cp*CDH, the substrate specificity of the recombinant *Cp*CDH was determined, and we observed a preference for disaccharides over monosaccharides. In particular, cellobiose was the most exclusive substrate for *Cp*CDH, followed by lactose. These findings are consistent with previous reports stating that adequate substrates for CDHs are disaccharides; cellobiose, lactose, and maltose, among others, but not monomeric sugars such as glucose, raffinose, or sorbose [9, 44]. The reason for this substrate specificity in class-I CDHs is thought to be due to the binding site on the non-reducing end of the substrate (B-site) on the sugar molecule. In class-I CDHs, highly conserved glutamic acid binds to the non-reducing end of the substrate, such as cellobiose, whereas in class-II CDHs, an asparagine in the same position fulfills a similar function [45]. In term of sucrose, the sugar structure was contained β-D-fructofuranosyl-(2→1)-α-D-glucopyranoside, that C1 of glucose structure is not available for *Cp*CDH. Furthermore, the mannobiose and xylobiose are presenting distinct stereochemistry and sugar structures divergent from that of glucose, exhibited unfamiliar behavior. As in CDH from *Phanerochaete chrysosporium*, substitution of amino acid residue E279Q resulted in the loss of substrate specificity [46]. The substrate specificity of *Cp*CDH is closely related to its catalytic efficiency, as shown in Table 2.

The *K*_m_ values of *Cp*CDH were similar for the disaccharide substrates cellobiose and lactose (2.24 and 6.93, respectively), with no significant difference to class-I CDHs. The substrate specificity of Class-I CDHs is strongly influenced by the presence of glutamic acid on the B-site of the substrate, although the amino acid sequence alignment of *Cp*CDH showed a lack of glutamic acid in the Thr^707^-Gly^713^ region (Fig. S1). On the other hand, the *Cp*CDH showed Arg^697^ and Glu^699^ instead, suggesting that Arg^697^ and Glu^699^ may contribute to the substrate specificity of *Cp*CDH. Class-I CDHs, which show a preference for cellobiose over lactose, with *K*_m_ values differing by 10- to 30-fold [30, 34, 47, 48], exhibit substrate specificity due to the highly conserved amino acid Glu^279^ in *Pc*CDH. This amino acid forms hydrogen bonds with the hydroxyl group at the C-2 and C-3 positions on the non-reducing end of cellobiose at subsite B [9, 36, 45]. Furthermore, Trp^349^ in *Cp*CDH, which is also shared among class-I CDHs as conserved tryptophane or phenylalanine amino acid, plays a role in holding the glycosyl moiety in place on the B-site and driving protein-carbohydrate complexation with various saccharide moieties (*e.g.*, cellobiose, lactose, and maltose) [9, 49]. The different key amino acids present in *Cp*CDH may contribute to its unique properties against substrate variety, thus emphasizing the novelty of the detected bacterial CDH, namely *Cp*CDH from *C. palmilytica* EW123. Due to limitation, the identity matrix comparison of *Cp*CDH against the characterized protein database yielded a correspondence of 21.96%. It is noteworthy that template-based structural modeling typically necessitates an identity matrix surpassing 40%, as advocated by Waterhouse *et al*. in 2018 [50], or a minimum of 30% identity matrix, as recommended by Fiser in 2010 [51], in order to achieve enhanced precision in protein structure prediction. Furthermore, the evaluation of protein structure prediction methods, as highlighted by the Critical Assessment of Structure Prediction (CASP), underscores the requirement for template-free modeling to manifest an overall topology alignment exceeding 60%, as outlined by Kryshtafovych *et al*. in 2019 [52].

In the context of this publication, our examination encompassed amino acid sequence homology analysis using the characterized protein database as a comparator. This analysis revealed the correct positioning of key amino acids, aligning with prior reports in the literature. Thus, the outcomes of the amino acid sequence alignment substantiate the robust foundation underpinning the entirety of our work.

Based on our current understanding, aldonic acids have become increasingly important in various fields, including food, medicine, and cosmetics. Recently, cellobionic and lactobionic acids, which are obtained by oxidizing cellobiose and lactose, respectively, have gained popularity and are widely used in various industrial applications [16, 17]. To demonstrate the enzymatic activity of *Cp*CDH in efficiently converting both sugars to sugar acids, the oxidation products were purified and analyzed. The results of HPLC and FT-IR analysis strongly supported the production of oxidative products from cellobiose and lactose by showing a clear differentiation in retention time and a dominance of carbonyl groups in both cellobionic and lactobionic acids (Fig. 4). These findings provide strong evidence that the enzymatic reaction and purification steps were successful in producing the desired oxidative products. Fig. S6 shows the purified oxidative products from cellobiose and lactose.

In conclusion, *Cp*CDH represents a unique and noteworthy bacterial CDH enzyme distinct from its fungal counterparts. The evolutionary relationship between bacterial and fungal CDHs enzymes, particularly in relation to the CYT domain, remains unclear. Nonetheless, *Cp*CDH is the first identified cellobiose dehydrogenase in the *Cellulomonas* genus and has the potential to generate sugar acids for various industrial applications. This discovery may contribute to a comprehensive understanding of the crude enzyme system of *C. palmilytica* EW123, which efficiently degrades untreated oil palm residues without product (cellobiose) inhibition of cellulolytic enzymes during bacterial growth.

## Supplemental Materials

Supplementary data for this paper are available on-line only at http://jmb.or.kr.



## Figures and Tables

**Table 1:** Substrate specificity of recombinant *Cp*CDH with various substrates.

Substrate	Activity (U mg^–1^)	
	ABTS	DCPIP
Glucose	0.05 ± 0.01	0.41 ± 0.01
Cellobiose	0.32 ± 0.03	3.43 ± 0.07
Lactose	0.23 ± 0.04	2.28 ± 0.12
Cellulose powder T20	ND	ND
PASC*	ND	ND

*Phosphoric acid swollen cellulose, ND: could not detect

**Table 2:** The catalytic efficiency of *Cp*CDH and other CDHs in both Class I and II toward substrates.

Enzyme	Substrates	*K*_m_ (mM)	*k*_cat_ (s^–1^)	*k*_cat_/*K*_m_ (M^–1^ s^–1^)	Reference
*Cp*CDH	Cellobiose	2.24	459.2	2.05 × 10^5^	This study
	Lactose	6.93	627.9	9.06 × 10^4^	
	Glucose	5783	8.17	141.32	
*Pc*CDH	Cellobiose	0.11	15.7	1.43 × 10^5^	[48]
	Lactose	1.1	13.4	1.22 × 10^4^	
	Glucose	1600	2.6	1.63	
*Cs*CDH	Cellobiose	0.14	25.1	1.79 × 10^5^	[34]
	Lactose	4.4	28.0	6.36 × 10^3^	
	Glucose	3300	3.8	1.15	
*Sr*CDH	Cellobiose	0.12	27.0	2.25 × 10^5^	[30]
	Lactose	2.0	26.0	1.30 × 10^4^	
	Glucose	1250	1.5	1.2	
*Nc*CDH	Cellobiose	0.16	53.0	3.31 × 10^5^	[45]
	Lactose	0.33	51.8	1.57 × 10^5^	
	Glucose	4000	37.7	9.435	

The cellobiose dehydrogenase enzyme source; *Cp*CDH from *Cellulomonas palmilytica*, *Pc*CDH from *Phanerochaete chrysosporium*, *Cs*CDH from *Ceriporiopsis subvermispora*, *Sr*CDH from *Sclerotium rolfsii*, and *Nc*CDH from *Neurospora crassa*.
