# Supplementary material for: Discovery of a Novel Cellobiose Dehydrogenase from Cellulomonas palmilytica EW123 and Its Sugar Acids Production
Source: J Microbiol Biotechnol. 2023 Oct 5;34(2):457–66. doi: 10.4014/jmb.2307.07004 (PMC10940743; doi:10.4014/jmb.2307.07004)
Supplement: Supplementary file 1 [file jmb-34-2-457-supple.pdf]

J. Microbiol. Biotechnol.
https://doi.org/10.4014/jmb.2307.07004

Supplementary Figures and Tables

Discovery of a novel cellobiose dehydrogenase from *Cellulomonas palmilytica* EW123 and its sugar acids production

Ake-kavitch Siriatcharanon[1], Sawannee Sutheeworapong[2], Sirilak Baramee[3], Rattiya Waeonukul[1,3], Patthra Pason[1,3], Akihiko Kosugi[4], Ayaka Uke[4], Khanok Ratanakhanokchai[1,3], Chakrit Tachaapaikoon[1,3 #]

```
CpCDH_AA3     AGGGRRPRSRSQAAQRSGTNAYEPCVESSTCPSARTTRCMVDRTGSTNRPGSASIPTSTS 60
AEO59298.1    ---------------------------------MKLLSRV-GATA--------------- 11
EAA28998.1    ---------------------------------MKVFTRI-GTIV--------------- 11
ADT70773.1    ---------------------------------MRPSSRFVGALA--------------- 12
ABS45567.2    ---------------------------------MRTSSRLIGALA--------------- 12
EAA27355.1    ---------------------------------MRTTSAFLSGLA--------------- 12
ADX41688.1    ------------------------------------------------------------ 0
CAA61359.1    ---------------------------------------MLGRS---------------- 5

CpCDH_AA3     GSSRAAAETRTRSQPPSRGRPSVPGPARSSRARPTRPGGCATN--RARFCAPHAAISSSS 118
AEO59298.1    ------------------------------LAATLSLQQCAAQMTEGTYTDEATGIQFKT 41
EAA28998.1    ------------------------------LATSLYLQQCSAQYINEQYTDPVNKITLST 41
ADT70773.1    ------------------------------AAASFLPSALAQNNAAVTFTDPDTGIVFNS 42
ABS45567.2    ------------------------------A--ALLPSALAQNNVPNTFTDPDSGITFNT 40
EAA27355.1    ------------------------------AVASLLSPAFAQ-TAPKTFTHPDTGIVFNT 41
ADX41688.1    -------------------------------------------QVAAPYVDSGNGFVFDG 17
CAA61359.1    ------------------------------LLALLPFVGLAFSQSASQFTDPTTGFQFTG 35

CpCDH_AA3     SRPST-SP--SGPTSHASSAVVHPDPAPTSSTVAPRGTSSRRSIAATVLGCEP----VEP 171
AEO59298.1    WTASE--------GAPFTFGLTLPADALE--------KDATEY--IGLLRCQITD-PASP 82
EAA28998.1    WRPDPGSNSGGGDAATYAFGLVLPPDALT--------KDANEY--IGLLRCDVGD-AASP 90
ADT70773.1    WGLANGAPQT---QGGFTFGVALPSDALT--------TDATEF--IGYLECASAD---NQ 86
ABS45567.2    WGLDEDSPQT---QGGFTFGVALPSDALT--------TDASEF--IGYLKCARND---ES 84
EAA27355.1    WSASD--SQT---KGGFTVGMALPSNALT--------TDATEF--IGYLECSSAKNGANS 86
ADX41688.1    ITDP---------VYHVSYGIVLPQAT-----------TSSEF--IGEIVAP-----LDA 50
CAA61359.1    ITDP---------VHDVTYGFVFPPLATS-------GAQSTEF--IGEVVAP-----IAS 72

CpCDH_AA3     RH---------------------------------------------------------S 174
AEO59298.1    SWCGISHGQSGQMTQALLLVAWASEDTVYTSFRYATGYTLPGLYTGDAKLTQIS-SSVSE 141
EAA28998.1    GWCGVSHGQSGQMTQSLLLMAWASKGQVFTSFRYASGYNVPGLYTGNATLTQIS-ATVNS 149
ADT70773.1    GWCGVSMG--GPMTNSLLITAWPHEDNVYTSLRFATGYAMPDVYSGDATITQIS-SSINA 143
ABS45567.2    GWCGISLG--GPMTNSLLITAWPHEDTVYTSLRFATGYAMPDVYEGDAEITQVS-SSVNS 141
EAA27355.1    GWCGVSLR--GAMTNNLLITAWPSDGEVYTNLMFATGYAMPKNYAGDAKITQIA-SSVNA 143
ADX41688.1    KWIGLALG--GAMIGDLLIVAWPNGNEIVSSTRYATAYQLPDVYEGP-TITTLPSSLVNS 107
CAA61359.1    KWIGIALG--GAMNNDLLLVAWANGNQIVSSTRWATGYVQPTAYTGTATLTTLPETTINS 130

CpCDH_AA3     GSARSSAACARCAGGRN--------STRGTARIAAATRSLVEGAPGSSSCSSCSCSSCSC 226
AEO59298.1    DSFEVLFRCENCFSWDQDGTKGNVSTSNGNLVL---GRAAAKDGVTGPTCPDTAEFGF-- 196
EAA28998.1    TQFELIYRCQDCFAWNQGGSKGSVSTSSGLLVL---GRAAAKGNLQNPTCPDKAIPGF-- 204
ADT70773.1    THFKLIFRCQNCLQWTHDGASGGASTSAGVLVL---GWVQAFPSPGNPTCPDQITLEQ-- 198
ABS45567.2    THFSLIFRCKNCLQWSHGGSSGGASTSGGVLVL---GWVQAFDDPGNPTCPEQITLQQ-- 196
EAA27355.1    THFTLVFRCQNCLSWDQDGVTGGISTSNKGAQL---GWVQAFPSPGNPTCPTQITLSQ-- 198
ADX41688.1    THWKFVFRCQNCTSWEGGGGIDP----TGTGVF---AWAYSSVGVDDPSDPNTTFQEH-- 158
CAA61359.1    THWKWVFRCQGCTEWNNGGGIDV----TSQGVL---AWAFSNVAVDDPSDPQSTFSEH-- 181

CpCDH_AA3     SSCSCSSCMAPPSPRARWGPPGSSGRRRAPHVAVAAHAVLTTDTRRV--TAPRTTT-TSS 283
AEO59298.1    ----------HDNGFGQWGAVLEG----ATSDSYEEWAKLATTTPETTCDGTGP------ 236
EAA28998.1    ----------HDNGFGQYGAPLEK----VPHTSYSAWASLATKTTTADCSGASDPVPTGS 250
ADT70773.1    ----------HNNGMGIWGAVMDSN---VANPSYTEWAAQATKTVEAECDGPSETDI--- 242
ABS45567.2    ----------HDNGMGIWGAQLNTD---AASPSYTDWAAQATKTVTGDCEGPTETSV--- 240
EAA27355.1    ----------HDNGMGQWGAAFDSN---IANPSYTAWAAKATKTVTGTCSGPVTTSI--- 242
ADX41688.1    ------------TDFGFFGINFPD----AQNSNYQNYLQGNAGTPPPTSTPSGPTT---- 198
CAA61359.1    ------------TDFGFFGIDYST-----DSANYQNYLNGDSGNPTTTSTKPTSTS---- 220
```

```
CpCDH_AA3      T----------PPATDAPSGGMLDADVVVVGSGFGGSVAALRLTEKGYRVLVVEAGRRFT 333
AEO59298.1     -----------GDKECVPAP-EDTYDYIVVGAGAGGITVADKLSEAGHKVLLIEKGPPST 284
EAA28998.1     EPPAEPTSTAEPVPVCTPAP-SKTYDYIIVGAGAGGIPIADKLSEAGKSVLLIEKGPPST 309
ADT70773.1     --------------VGVPVPTGTTFDYIVVGGGAGGIPTADKLSEAGKSVLLIEKGIAST 288
ABS45567.2     --------------VGVPVPTGVSFDYIVVGGGAGGIPAADKLSEAGKSVLLIEKGFAST 286
EAA27355.1     --------------AATPVPTGVSFDYIVVGGGAGGIPVADKLSESGKSVLLIEKGFAST 288
ADX41688.1     -----------TSKPTGPTASATPYDYIIVGAGPGGIIAADRLSEAGKKVLLLERGGPST 247
CAA61359.1     -----------SSVTTGPTVSATPYDYIIVGAGPGGIIAADRLSEAGKKVLLLERGGPST 269

                                       •
CpCDH_AA3      DETLPR-----------TSWDVRR---FLWAPRLGCTGIQRIHVLPDVVVLAGAGVGGGS 379
AEO59298.1     GLWNGTMKPEWLESTDLTRFDVPGLCNQIWVDSAG-------IACTDTDQMAGCVLGGGT 337
EAA28998.1     GRWKGTMKPEWLQGTNLTRFDVPGLCNQIWVDSAG-------IACTDTDQMAGCVLGGGT 362
ADT70773.1     AEHGGTLGPEWLEGNDLTRFDVPGLCNQIWVDSKG-------IACEDTDQMAGCVLGGGT 341
ABS45567.2     ANTGGTLGPEWLEGHDLTRFDVPGLCNQIWVDSKG-------IACEDTDQMAGCVLGGGT 339
EAA27355.1     GEHGGTLKPEWLNNTSLTRFDVPGLCNQIWKDSDG-------IACSDTDQMAGCVLGGGT 341
ADX41688.1     AETGGTYYAPWAKSQNLTKFDIPGLFESMFTDPNP------WWWCKDTNFFAGCLLGGGT 301
CAA61359.1     KQTGGTYVAPWATSSGLTKFDIPGLFESLFTDSNP------FWWCKDITVFAGCLVGGGT 323

CpCDH_AA3      LVYANTLYEPERDEFWDD--PQWAGITDWRDELAPHYDQARRMLGVVDNPTVTPADEVVR 437
AEO59298.1     AVNAGLWWKPHPADWDENFPEGWK--S--SDLAD----ATERVFKRIPGTS-HPSQDGKL 388
EAA28998.1     AVNAGLWWKPHPQDWNYNFPEGWK--S--RDTVP----ATNRVFGRIPGTW-HPSQNGKL 413
ADT70773.1     AVNAGLWFKPYSLDWDYLFPSGWK--Y--RDIQA----AIGRVFSRIPGTD-APSTDGKR 392
ABS45567.2     AVNAGLWFKPYSLDWDYLFPDGWK--Y--NDVQP----AINRALSRIPGTD-APSTDGKR 390
EAA27355.1     AINAGLWYKPYTKDWDYLFPSGWK--G--SDIAG----ATSRALSRIPGTT-TPSQDGKR 392
ADX41688.1     SVNGGALYWLPSDADFST--ANGWP--TSWGNHAP----YTSKLKQRLPSTD-HPSTDGKR 352
CAA61359.1     SVNGGALYWYPNDGDFSS--SVGWP--SSWTNHAP----YTSKLSSRLPSTD-HPSTDGQR 374
                                                                          :
CpCDH_AA3      AAARDLGVGASFRLAPVGVVFGRDGRLEPG--QAVPDPFFGGVGPDRRGCTQCGACMTGC 495
AEO59298.1     Y------RQEGFEVISKGLANAGWKEISANEAPSEKNHTYAHTEFM---------FSGGE 433
EAA28998.1     Y------RQEGFNVLASGLSKSGWKEVIPNDAYNQKNHTFGHSTFM---------FAKGE 458
ADT70773.1     Y------YQQGFDVLAGGLSAGGWNKVTANSSPDKKNRTFSNAPFM---------FSGGE 437
ABS45567.2     Y------YQEGFEVLSKGLAAGGWTSVTANNAPDKKNRTFAHAPFM---------FAGGE 435
EAA27355.1     Y------LQQGFEVLANGLKASGWKEVDSLKDSEQKNRTFSHTSYM---------YINGE 437
ADX41688.1     Y------LEQSATVVSQLLQGQGYQQITINDNPDSKDHVFGYSAFD---------FINGQ 397
CAA61359.1     Y------LEQSFNVVSQLLKGQGYNQATINDNPNYKDHVFGYSAFD---------FLNGK 419

CpCDH_AA3      RFGAKNTLTTNYLALAERAGARVVPLTTVTSVRPA-ADGTWQVDV-----AATGHPRRGR 549
AEO59298.1     RGG----PLATYLASAAERSNFN--LWLNTAVRRAVRSGSKVTGVELECLTDGGFSGTVN 487
EAA28998.1     RGG----PLATYLVTAVARKQFT--LWTNVAVRRAVRNGSRITGVELECLTDGGLSGTVN 512
ADT70773.1     RGG----PLATYLTSAKKRSNFN--LWLNTSVKRVIREGGHVTGVEVEPFRTGGYQGIVN 491
ABS45567.2     RNG----PLGTYFQTAKKRNNFD--VWLNTSVKRVIREGGHITGVEVEPFRDGGYEGIVP 489
EAA27355.1     RGG----PLATYLVSAKKRSNFK--LWLNTAVKRVIREGGHITGVEVEAFRNGGYSGIIP 491
ADX41688.1     RAG----PVATYFQTASARSNFV--YKDFTLVSQVLRNGSTITGVRTNNTA-LGPDGIVP 450
CAA61359.1     RAG----PVATYLQTALARPNFT--FKTNVMVSNVVRNGSQILGVQTNDPT-LGPNGFIP 472

CpCDH_AA3      RTLRTGQVVLAAGAWGTQALLHGLKADGVLPELSDRLGHLTRTNSEALGGAVARWRDRRT 609
AEO59298.1     LNE-GGGVIFSAGAFGSAKLLLRS---GIGPEDQLEIVASS-KDGETF-TPKDEWINLPV 541
EAA28998.1     VTPNTGRVIFAAGTFGSAKLLLRS---GIGPTDQLEIVKGS-TDGPTF-ISKDQWINLPV 567
ADT70773.1     VTAVSGRVVLSAGTFGSAKILLRG---GIGPADQLEVVKASKIDGPTM-ISNASWIPLPV 547
ABS45567.2     VTKVTGRVILSAGTFGSAKILLRS---GIGPEDQLEVVAASEKDGPTM-IGNSSWINLPV 545
EAA27355.1     VTNTTGRVVLSAGTFGSAKILLRS---GIGPKDQLEVVKAS-ADGPTM-VSNSSWIDLPV 546
ADX41688.1     LNP-NGRVILAAGSFGTPRILFQS---GIGPTDMIQTVQSNPTAAANL-PPESEWINLPV 505
CAA61359.1     VTP-KGRVILSAGAFGTSRILFQS---GIGPTDMIQTVQSNPTAAAAL-PPQNQWINLPV 527
```

```
CpCDH_AA3       APDLTQGVAITSSVWLDERTHLEPVRYGRGSNLMGLLSTVLTSGRTRAGDSGAGDSGAGG 669
AEO59298.1      GHNLIDHLNTDLI-----ITHPDVVFYD----FYAAWDEPITEDKEAYLNSRSGILAQAA 592
EAA28998.1      GYNLMDHLNTDLI-----ITHPDVVFYD----FYEAWNTPIEGDKSAYLQNRSGILAQAA 618
ADT70773.1      GYNLDDHLNTDTV-----ITHPDVAFYD----FYEAWNTPIEADKNSYLSSRTGILAQAA 598
ABS45567.2      GYNLDDHLNTDTV-----ISHPDVVFYD----FYEAWDDPIESDKNSYLESRTGILAQAA 596
EAA27355.1      GHNLVDHTNTDTV-----IQHNNVTFYD----FYKAWDNPNTTDMNLYLNGRSGIFAQAA 597
ADX41688.1      GQGVSDNPSINLV-----FTHPSIDAYEN---WADVWSNPRPADAQQYLQSRSGVFAGAS 557
CAA61359.1      GMNAQDNPSINLV-----FTHPSIDAYEN---WADVWSNPRPADAAQYLANQSGVFAGAS 579

                                                     • •
CpCDH_AA3       PGAGGSGAGGSGAGGARPAGGSGAGGARVERALRWVGTVARHPG---------QV--ASL 718
AEO59298.1      PNIGPM--------MW----DQVTPSDGITRQFQWTCRVEGDSS----KTNSTHAMTLSQ 636
EAA28998.1      PNIGPL--------MW----DELKGSDNIIRTLQWTARVEGSDQ----YTTSKHAMTLSQ 662
ADT70773.1      PNIGPM--------MW----EEIKGADGIVRQLQWTARVEGSFD-----TPNGQAMTISQ 641
ABS45567.2      PNIGPM--------FW----EEIVGADGIVRQLQWTARVEGSLG-----APNGHTMTMSQ 639
EAA27355.1      PNIGPL--------FW----EEITGADGIVRQLHWTARVEGSFE-----TPDGYAMTMSQ 640
ADX41688.1      PKL--N--------FW----RAYGGSDGKTRYAQGTVRPGAASVNTSVAYNASQIFTITV 603
CAA61359.1      PKL--N--------FW----RAYSGSDGFTRYAQGTVRPGAASVNSSLPYNASQIFTITV 625

                •
CpCDH_AA3       LLGIGSWSQRTVIGLV----MQTGGASIVVRP---RRTWRGTVRLTSTPGEGEPNPTWIP 771
AEO59298.1      YLGRGVVSRGR-MGITSGLSTTVAEHPYLHNNGDLEAVIQGIQNVVD-ALSQVADLEWVL 694
EAA28998.1      YLGRGVVSRGR-MAISSGLDTNVAEHPYLHNDVDKQTVIQGIKNLQA-ALNVIPNLSWVL 720
ADT70773.1      YLGRGATSRGR-MTITPSLTTVVSDVPYLKDPNDKEAVIQGIVNLQN-ALKNVAGLTWTY 699
ABS45567.2      YLGRGATSRGR-MTITPSLTTIVSDVPYLKDPNDKEAVIQGIINLQN-ALQNVANLTWLF 697
EAA27355.1      YLGRGATSRGR-MTLSPTLNTVVSDLPYLKDPNDKAAVVQGIVNLQK-ALANVKGLTWAY 698
ADX41688.1      YLSEGITSRGR-LGVDAALNMKAITTPWLTDPVDKTILLQALHDVVS-NINNVPGLTLIT 661
CAA61359.1      YLSTGIQSRGR-IGIDAALRGTVLTPPWLVNPVDKTVLLQALHDVVS-NIGSIPGLTMIT 683

                                             ••
CpCDH_AA3       QAHAAYRAMARRLGGFAASSLAEVVDVPMTAHFIGGCTIGRT-----RDDGVVDPYHRVH 826
AEO59298.1      PPPD------GTVADYVNSLI-VSPANRRANHWMGTAKLGTDDGRS-GGTSVVDLDTKVY 746
EAA28998.1      PPPN------TTVESFINNMI-VSPSNRRSNHWMGTAKLGKDDGRT-GGSAVVDLNTKVY 772
ADT70773.1      PNSS------ITPREYVDNMV-VSPSNRRANHWMGTAKIGTDDGRLAGGSAVVDLNTKVY 752
ABS45567.2      PNST------ITPREYVESMV-VSPSNRRSNHWMGTNKLGTDDGRK-GGSAVVDLDTRVY 749
EAA27355.1      PSAN------QTAADFVDKQP-VTYQSRRSNHWMGTNKMGTDDGRS-GGTAVVDTNTRVY 750
ADX41688.1      PDHT------QTLEQYVAAY---DPATMCSNHWVGAAKIGSS-----PSTAVVDENTKVF 707
CAA61359.1      PDVT------QTLEEYVDAY---DPATMNSNHWVSSTTIGSS-----PQSAVVDSNVKVF 729
                                               ▲

CpCDH_AA3       GYPGLHVLDGSTISANLGVNPSLTITAQAERACALWPNRGEPDPRPAPGRPYARVAPVAP 886
AEO59298.1      GTDNLFVVDASVFPGMSTGNPSAMIVIVAEQAAQRILALRS------------------- 787
EAA28998.1      GTDNLFVVDASIFPGMTTGNPSAMIVIASEHAAQKILALKP------------------- 813
ADT70773.1      GTDNLFVVDASIFPGTPTTNPSAYIVTAAEHASQRILGLAA------------------- 793
ABS45567.2      GTDNLFVIDASIFPGVPTTNPTSYIVVAAEHASSRILALPD------------------- 790
EAA27355.1      GTDNLYVVDASIFPGVPTTNPTAYIVVAAEHAAAKILAQPA------------------- 791
ADX41688.1      NTDNLFIVDASIIPSLPVGNPHGALMSAAEQAAAKILALAG------------------- 748
CAA61359.1      GTNNLFIVDAGIIPHLPTGNPQGTLMSAAEQAAAKILALAG------------------- 770
                                   ▲

CpCDH_AA3       ARPVVPAHAPAALRLTPVRPPGPPGPAGGSGGD---VGEM-------------------  923
AEO59298.1      -----------------------------------------------------------  787
EAA28998.1      ---------------------VPSLPGG--NGKGKWRR---------------------  828
ADT70773.1      ---------------------PKPVGKWGQCGGRQWTGSFQCVSGTKCEVVNEWYSQCL  831
ABS45567.2      ---------------------LEPVPKYGQCGGREWTGSFVCADGSTCEYQNEWYSQCL  828
EAA27355.1      ---------------------NEAVPKWGWCGGPTYTGSQTCQAPYKCEKQNDWYWQCV  829
ADX41688.1      ---------------------GP------------------------------------  750
CAA61359.1      ---------------------GP------------------------------------  772
```

Fig. S1. The full sequence alignment of *Pc*CDH with other characterized cellobiose dehydrogenases. The font character and symbol were illustrated as follows; bold alphabet: signal peptide of Sec secretion system, underline alphabet: linker between two domains, italic alphabet: CBM domain, gray color fill: FAD region, solid triangle: catalytic amino acid. The sequence codes were retrieved as follow; AEO59298: *Myriococcum thermophilum*, EAA28998 and EAA27355: *Neurospora crassa*, ADT70773: *Chetomium atrobrunneum*, ABS45567: *Crassicarpon hotsonii*, ADX41688: *Trametes cinnabarina* and CAA61359: *Phanerochaete chrysosporium*.

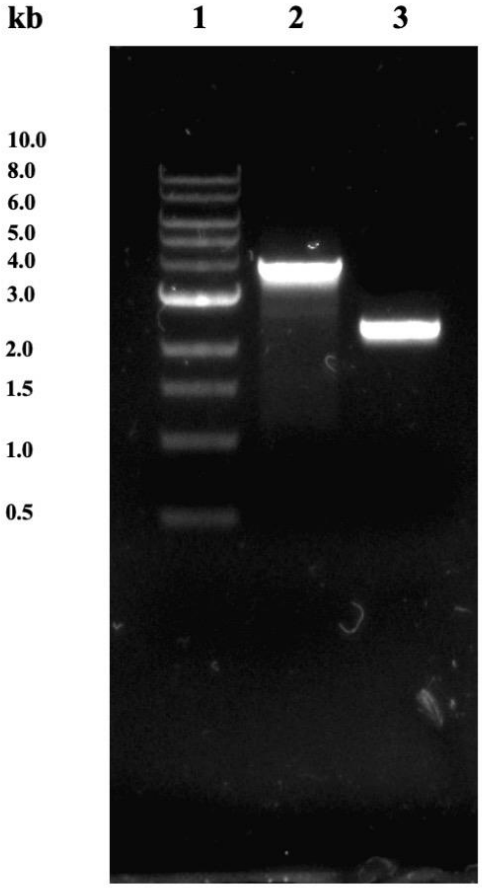

Fig. S2. The PCR product on agarose gel. Lane 1 is 1 kb ladder (neb), lane 2 is the nested-PCR product and lane 3 is the *cdh* gene.

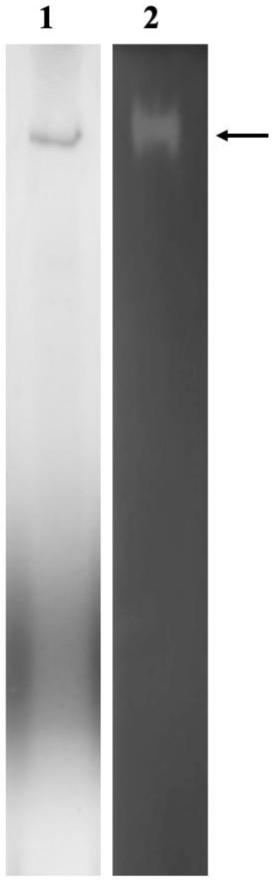

Fig. S3. The PAGE analysis of recombinant *Cp*CDH and in-gel activity. Each lane was described as follows: Lane 1 is purified recombinant *Cp*CDH, and Lane 2 is the zymogram of CDH activity. The target recombinant *Cp*CDH was indicated by an arrow.

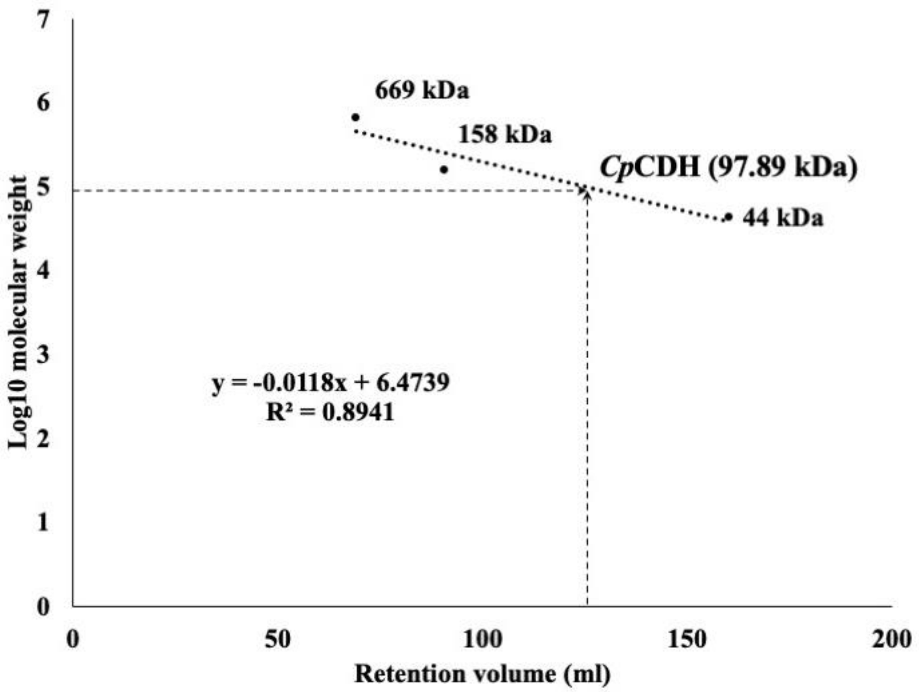

Fig. S4. Gel filtration chromatography calibration equation generated by standard protein calibration kit (Gel Filtration Calibration Kits (HMW), Cytiva). The purified recombinant *Cp*CDH was used as the subject.

```
Name     Amino acid sequence                Percentage identity

CpCDH    VVVGSGFGGSVAALRLTEKGYRVLVVEAGRRFT 100.00
ADX41688 IIVGAGPGGIIAADRLSEAGKKVILLERGGPST  45.45 100.00
AEO59298 IVVGAGAGGITVADKLSEAGHKVLLIEKGPPST  45.45  69.70 100.00
CAA61359 IIVGAGPGGIIAADRLSEAGKKVLLLERGGPST  48.48  96.97  72.73 100.00
EAA28998 IIVGAGAGGIPIADKLSEAGKSVLLIEKGPPST  42.42  72.73  84.85  75.76 100.00
ADT70773 IVVGGGAGGIPTADKLSEAGKSVLLIEKGIAST  45.45  63.64  78.79  66.67  84.85 100.00
ABS45567 IVVGGGAGGIPAADKLSEAGKSVLLIEKGFAST  48.48  66.67  78.79  69.70  84.85  93.94 100.00
EAA27355 IVVGGGAGGIPVADKLSESGKSVLLIEKGFAST  45.45  60.61  78.79  63.64  81.82  90.91  93.94  100.00
         ::**.* **   * :*:* *  *:::* *   *


CpCDH    LAGAGVGGGSLVYANTLYEPERDEFW        100.00
ADX41688 FAGCLLGGGTSVNGALYWLPSDADFS         30.77 100.00
AEO59298 MAGCVLGGGTAVNAGLWWKPHPADWD         30.77  57.69 100.00
CAA61359 FAGCLVGGGTSVNGALYWYPNDGDFS         34.62  84.62  50.00 100.00
EAA28998 MAGCVLGGGTAVNAGLWWKPHPQDWN         30.77  53.85  92.31  50.00 100.00
ADT70773 MAGCVLGGGTAVNAGLWFKPYSLDWD         30.77  50.00  84.62  46.15  80.77 100.00
ABS45567 MAGCVLGGGTAVNAGLWFKPYSLDWD         30.77  50.00  84.62  46.15  80.77 100.00 100.00
EAA27355 MAGCVLGGGTAINAGLWYKPYTKDWD         30.77  46.15  80.77  42.31  76.62  84.62  84.62 100.00
         :**. :***: : .   : *   ::


CpCDH    GQVVLAAGAWGTQALLHGLKAD            100.00
ADX41688 GRVILAAGSFGTPRILFQSGIG             40.91 100.00
AEO59298 GGVIFSAGAFGSAKLLLRSGIG             36.36  54.55 100.00
CAA61359 GRVILSAGAFGTSRILFQSGIG             40.91  86.36  63.64 100.00
EAA28998 GRVIFAAGTFGSAKLLLRSGIG             36.36  63.64  86.36  59.09 100.00
ADT70773 GRVVLSAGTFGSAKILLRGGIG             36.36  59.09  72.73  63.64  77.27 100.00
ABS45567 GRVILSAGTFGSAKILLRSGIG             31.82  68.18  81.82  72.73  86.36  90.91 100.00
EAA27355 GRVVLSAGTFGSAKILLRSGIG             36.36  63.64  77.27  68.18  81.82  95.45  95.45 100.00
         * *:::**::*:  :*     .
```

Fig. S5. The multiple sequence alignment of FAD regions and its percentage identity matrix.

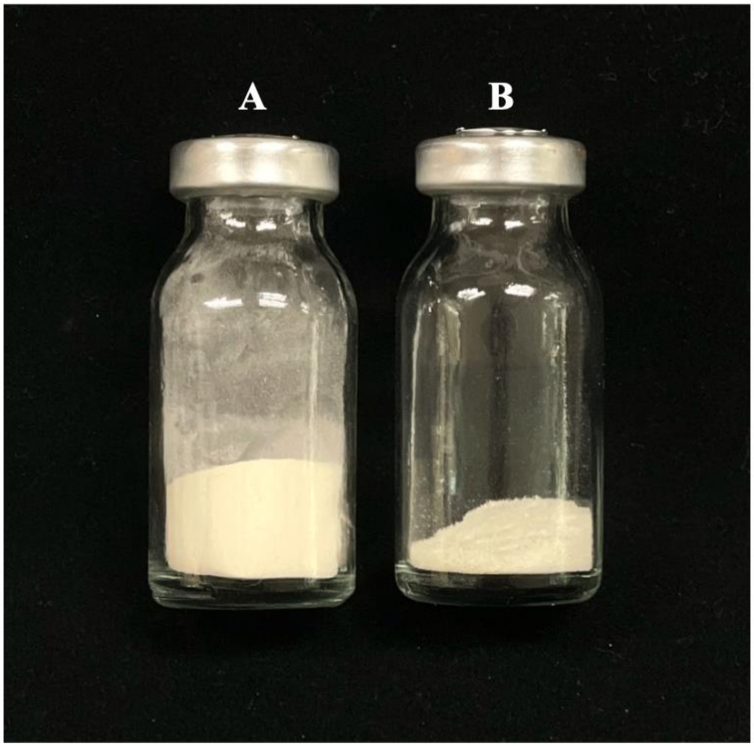

Fig. S6. Purified oxidative products from cellobiose (A) and lactose (B) that were used for FT-IR analysis.

Table S1. The TD-PCR thermocycle program in this study.

Step	Phrase	Temperature (°C)	Time
1	Initial denaturing	98	0:30
2	Denature	98	0:10
3	Anneal	72-60[a]	0:20
4	Extension	72	1:55[b] or 1:24[c]
	Repeat steps 2-4 for 10 cycles[*]		
5	Denature	98	0:10
6	Anneal		0:20
7	Extension	72	1:55[b] or 1:24[c]
	Repeat steps 5-7 for 20 cycles[*]		
8	Final extension	72	2:00
9	Final hold	4	∞

[a] The annealing temperature is reduced by 1.2°C for each cycle, [b] for nested-PCR amplification, [c] for gene specific amplification, [*] total thermocycle should not be exceed than 30 cycles because G+C content of *Cp*CDH was highly above the recommendation range.

Table S2 The amino acid composition and pI value in CDHs.

Amino acid residue	Amino acid composition in CDHs (%)							
	***Cp*CDH**	**AEO59298**	**EAA28998**	**ADT70773**	**ABS45567**	**EAA27355**	**ADX41688**	**CAA61359**
**Ala (A)**	12.8	9.5	8.2	9.5	7.9	9.7	9.5	8.8
**Arg (R)**	11.3	3.3	3.7	3.5	3.6	3.0	2.5	2.5
**Asn (N)**	1.5	3.9	5.7	5.3	5.1	5.8	6.0	6.2
**Asp (D)**	4.0	6.6	5.0	5.5	6.5	5.2	5.6	5.1
**Cys (C)**	2.5	1.7	1.4	1.6	1.6	1.6	0.7	0.5
**Gln (Q)**	1.8	3.3	4.0	3.6	3.3	3.7	4.3	4.5
**Glu (E)**	2.9	5.8	2.9	3.5	4.6	2.7	2.4	1.7
**Gly (G)**	12.9	10.8	10.9	11.6	11.4	10.7	10.0	9.8
**His (H)**	2.1	1.8	1.7	1.2	1.4	1.4	1.5	1.3
**Ile (I)**	1.7	3.9	4.8	4.9	4.8	4.1	5.5	4.9
**Leu (L)**	6.0	8.1	7.9	6.5	7.2	6.5	6.7	7.3
**Lys (K)**	0.3	3.4	4.6	3.4	3.0	4.7	2.7	2.3
**Met (M)**	1.1	1.7	1.7	1.8	1.6	1.8	0.8	1.0
**Phe (F)**	1.3	3.3	2.9	3.5	3.5	3.3	4.1	4.7
**Pro (P)**	8.8	4.7	6.4	5.7	5.8	5.1	7.7	7.0
**Ser (S)**	9.6	7.6	7.5	7.5	7.6	7.7	8.3	9.5
**Thr (T)**	8.3	8.9	8.5	8.4	8.3	10.0	9.1	10.0
**Trp (W)**	1.6	2.7	2.7	2.8	2.7	2.9	2.4	2.5
**Tyr (Y)**	1.2	2.4	3.0	2.9	3.0	3.4	3.7	3.2
**Val (V)**	8.2	6.5	6.6	7.5	7.1	6.8	6.7	7.3
**Total negatively charged residues**	64	98	65	75	92	65	60	52
**Total positively charged residues**	107	53	69	57	55	64	39	37
**Theoretical pI**	10.86	4.58	8.28	5.10	4.65	6.86	4.86	5.06

The sequence codes were retrieved as follow; AEO59298: *Myriococcum thermophilum*, EAA28998 and EAA27355: *Neurospora crassa*, ADT70773: *Chetomium atrobrunneum*, ABS45567: *Crassicarpon hotsonii*, ADX41688: *Trametes cinnabarina* and CAA61359: *Phanerochaete chrysosporium*.
